# The breast cancer immune microenvironment is modified by neoadjuvant chemotherapy

**DOI:** 10.1038/s41598-022-12108-5

**Published:** 2022-05-13

**Authors:** Claudia Urueña, Paola Lasso, David Bernal-Estevez, Diego Rubio, Ana Janeth Salazar, Mercedes Olaya, Alfonso Barreto, Mauricio Tawil, Lilian Torregrosa, Susana Fiorentino

**Affiliations:** 1 Grupo de Inmunobiología y Biología Celular, Unidad de Investigación en Ciencias Biomédicas, Facultad de Ciencias, Pontificia Universidad Javeriana, Carrera 7a. No. 43-82, Ed. 50, Lab. 101, C.P. 110211 Bogotá, Colombia; 2 Grupo de Investigación en Inmunología y Oncología Clínica, Fundación Salud de los Andes, Bogotá, Colombia; 3 Departamento de Patología, Hospital Universitario San Ignacio, Bogotá, Colombia; 4 Departamento de Cirugía y Especialidades, Hospital Universitario San Ignacio, Centro Javeriano de Oncología, Facultad de Medicina, Pontificia Universidad Javeriana, Bogotá, Colombia

**Keywords:** Breast cancer, Cancer microenvironment, Tumour immunology

## Abstract

Neoadjuvant chemotherapy (NAT) in breast cancer (BC) has been used to reduce tumor burden prior to surgery. However, the impact on prognosis depends on the establishment of Pathological Complete Response (pCR), which is influenced by tumor-infiltrating lymphocyte levels and the activation of the antitumor immune response. Nonetheless, NAT can affect immune infiltration and the quality of the response. Here, we showed that NAT induces dynamic changes in the tumor microenvironment (TME). After NAT, an increase of regulatory T cells and a decrease of CD8^+^ T cells was found in tumor, correlated with the presence of metastatic cells in lymph nodes. In addition, an increase of polymorphonuclear myeloid-derived suppressor like cells was found in luminal patients post-NAT. pCR patients showed a balance between the immune populations, while non-pCR patients presented an inverse relationship in the frequency of CD68^+^ versus CD3^+^, CD8^+^, and CD20^+^ cells. Moreover, activated T cells were found in peripheral blood, as well as an increase in T cell clonality with a lower diversity post-NAT. Overall, these results shown that NAT induces an activation of immune response, however, a balance in the TME seems to be related to a better antigenic presentation and therefore a better response to treatment.

## Introduction

NAT is extensively used in early-stage BC and locally advanced BC as it helps to provide greater chances for breast conserving surgery^1,2^. Patients showing a pCR to NAT or only minimal residual disease, as defined by the residual cancer burden (RCB 0), may experience prolonged disease-free survival^3^ and might be related to intrinsic antitumor immune response activation. The role of the immune system in cancer is gradually being elucidated. In fact, in BC, an elevated immune infiltrate with a greater diversity in the response of T cells has been associated with a better outcome^4^ and with better survival in Her-2-negative patients, particularly when the infiltrate is mainly CD8^+^ T cells^5,6^. In addition, high immune infiltration of T cells has been associated with an increase in the response to NAT^7,8^ and with a decrease in tumor proliferation measured as a reduction in intratumoral Ki67^9,10^. However, tumor immunity is governed in a complex network between antitumoral and protumoral immune cells. Thus, in the TME of different cancers, including BC, increased levels of Tregs^11^ and myeloid-derived suppressor cells (MDSCs)^12^ have been described. Tregs and MDSCs represent two immunosuppressive cell populations that are important in the establishment and maintenance of cancer immune tolerance, and their abundance has been reported to be associated with a poor response to NAT and a poor clinical outcome in BC patients (BCP)^13,14^.

The type of immune response identified by immunological clusters has recently been related to pCR. Cluster C, described as the cluster with the highest immune infiltrate, present in estrogen receptor (ER)-negative and basal-like patients, is the cluster that best responds to chemotherapy, while cluster B, composed mainly of a protumorigenic infiltrate such as M2 macrophages, presents a lower response. Interestingly, there is an association between the immune cluster and the number of stem cell/epithelial–mesenchymal transition (EMT)-related gene signatures^15^. In a previous study by our group, we observed an accumulation of tumor cells of the ALDH^+^ stem phenotype in response to NAT in all molecular subtypes except Her-2^16^. This finding suggests that the intrinsic differences of tumors may also play a role in the control of the microenvironment, as previously reported^17–19^. To date, several reports have linked the presence or absence of certain cell types in the TME with tumor stages, prognosis, and patient survival^20,21^, however, the effects of NAT on the TME are still being studied. Some studies have evaluated the number of T cells in tumor tissues, but their clonality and changes in the T cell repertoire have not been well investigated.

In this study, we evaluated the effect of NAT on the TME by analyzing the immune cell populations present in the tumor as well as the diversity of the T cell response in blood and tumor before and after NAT in BCP.

## Materials and methods

### Healthy donors and breast cancer patients

The study was conducted with ethical approval from Hospital Universitario San Ignacio and Centro Javeriano de Oncología, Bogotá D.C., Colombia (Act 16/2016) and performed in compliance with Helsinki declaration. All patients provided written informed consent to participate in the study before any sample collection. Samples from healthy donors (HD) were collected from patients with benign breast pathologies (fibroadenoma) or reduction mammoplasty. Samples from BCP were collected from patients who received NAT prior to surgery. The inclusion of the patients in the study was carried out between 2016 and 2019. The patients were classified according to the molecular subtypes reported by WHO^22^ as Luminal A patients (ER+, PR ≥ 20%, Her2- and Ki67low), Luminal B patients (ER+, PR < 20% or Her2+/or Ki67high), HER2 (ER-, PR-, Her2+), Basal like (ER-, PR-, HER2-). Table 1 shows the clinical and pathological characteristics of all participating subjects.

**Table 1:** Clinicopathological characteristics of patients with breast cancer.

Characteristics	Luminal A (n = 11)	Luminal B (n = 15)	Her2 (n = 2)	Triple Negative (n = 5)	HD (n = 10)
**Age (years)**					
< 40	1 (9.1)	0 (0)	0 (0)	2 (40.0)	5 (50.0)
40–49	1 (9.1)	3 (20.0)	1 (50.0)	1 (20.0)	5 (50.0)
50–65	4 (36.3)	5 (33.3)	0 (0)	2 (40.0)	0 (0)
> 65	5 (45.4)	7 (46.7)	1 (50.0)	0 (0)	0 (0)
**Lymph nodes**					
Negative	9 (81.8)	8 (53.3)	1 (50.0)	3 (60.0)	
Positive	2 (18.1)	7 (46.7)	1 (50.0)	2 (40.0)	
**TNM stage(AJCC)**					
I	0 (0)	1 (6.7)	0 (0)	1 (20.0)	
II	7 (63.6)	11 (73.3)	0 (0)	2 (40.0)	
III	4 (36.3)	3 (20.0)	2 (100)	2 (40.0)	
IV	0 (0)	0 (0)	0 (0)	0 (0)	
**Ki67**					
< 20%	10 (90.0)	0 (0)	1 (50.0)	1 (20.0)	
> 20%	1 (0.09)	15 (100)	1 (50.0)	4 (40.0)	
**Neoadjuvant chemotherapy**					
AC	0(0)	2 (13.3)	1 (50.0)	1 (20.0)	
AC + TX	8 (72.7)	9 (60.0)	1 (50.0)	2 (40.0)	
Non therapy	3 (27.2)	4 (26.7)	0 (0)	2 (40.0)	
**Pathological response**					
pCR	0(0)	5 (33.3)	1 (50.0)	3 (60.0)	
Non-pCR	11 (100)	10 (66.6)	1 (50.0)	2 (40.0)	

HER2, human epidermal growth factor receptor 2; AC, Anthracyclines-Cyclophosphamide; TX, taxane; pCR, pathological complete response; Non-pCR, non pathological.

### Peripheral blood processing

**Figure 1:**
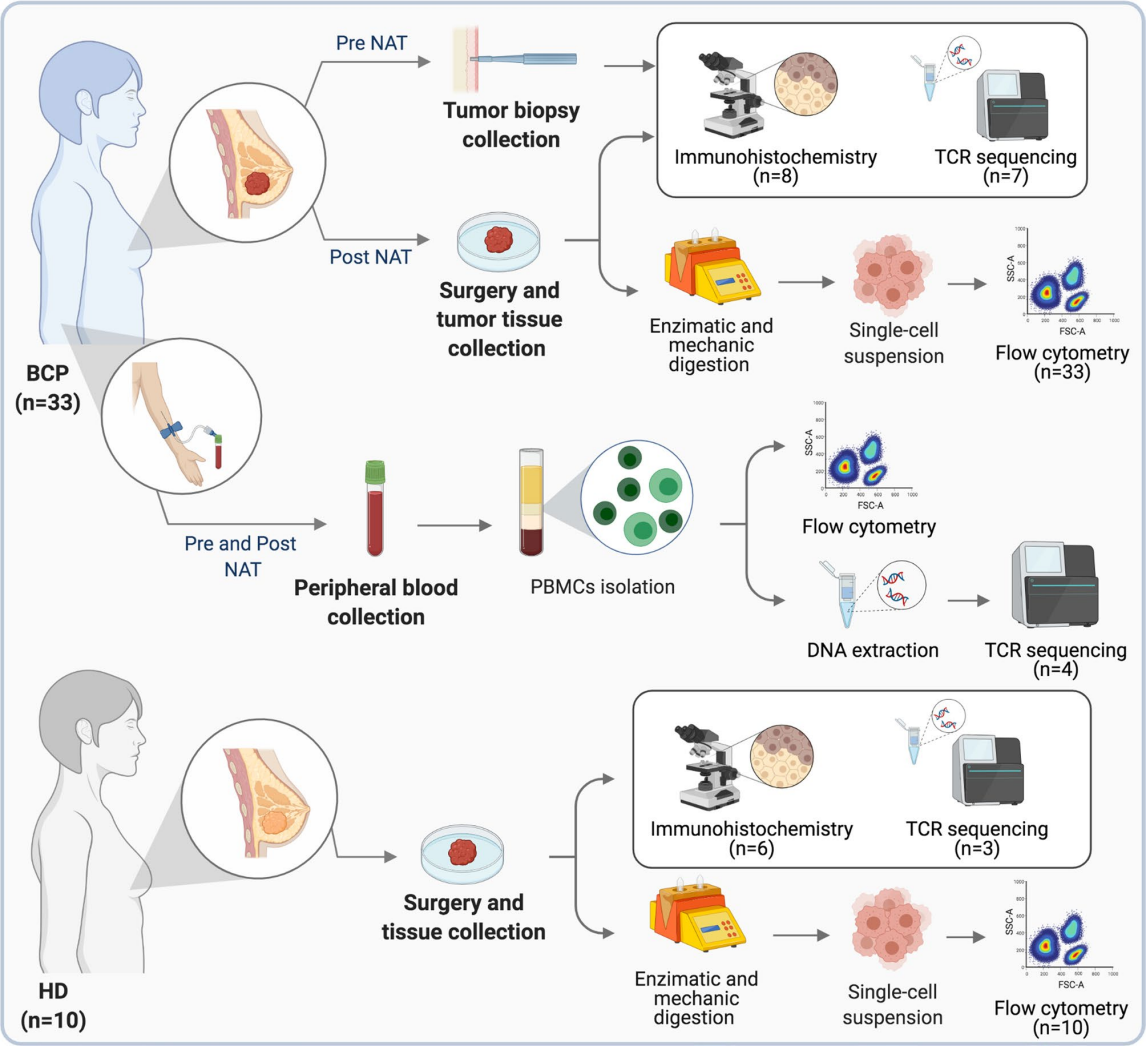
Experimental design. Breast cancer patients (BCP) and healthy donors (HD) were included in the study. From the patient group, biopsies and tumors were taken before and after NAT, respectively, and IHC and TCR sequencing were performed. Additionally, a section of the tumor sample was processed by enzymatic and mechanical digestion and used to evaluate different cell populations by flow cytometry. A blood sample was also taken from each patient before and after NAT to assess the activation status of T cells by flow cytometry and for TCR sequencing. From the HD group, breast tissue was taken during surgery for IHC, TCR sequencing and flow cytometry. This figure was created using BioRender (https://biorender.com/).

Peripheral blood was collected prior to NAT and post-NAT at the time of surgery (Fig. 1). Peripheral blood mononuclear cells (PBMCs) were isolated by density-gradient centrifugation using Ficoll-Paque PREMIUM (GE Healthcare, Chicago, Illinois, USA). A total of 1 × 10^7^ cells were cryopreserved in liquid nitrogen in freezing media (RPMI-1640 50%, FBS 40% and 10% DMSO) until use for flow cytometry characterization. A total of 1 × 10^6^ cells were used for DNA extraction with an UltraClean DNA Blood Isolation kit (MOBIO laboratories, Carlsbad, CA, USA) according to the manufacturer’s protocol for subsequent TCR sequencing (Fig. 1).

### Tissue sample collection and processing

Prior to NAT, a tissue sample was removed during a biopsy, and then, the tissue was formalin-fixed and paraffin embedded for immunohistochemistry (IHC) and/or TCR sequencing (Fig. 1). After NAT, the tissue was collected during surgery and divided into two pieces, one for flow cytometry characterization and the other for IHC and TCR sequencing (Fig. 1). For flow cytometry, tissues were minced into small pieces and dissociated into single cells by a combined mechanical/enzymatic process using the gentleMACS Dissociator (Miltenyi Biotec, Bergisch Gladbach, Germany) and the Tumor Dissociation kit, human (Miltenyi Biotec), according to the manufacturer’s instructions. Dissociated cells were collected after passage through a 100-μm nylon mesh filter and cultured at 37 °C and 5% CO_2_ overnight. Then, flow cytometry characterization was performed. For IHC and TCR sequencing, the tissue was formalin-fixed and paraffin embedded until processing.

### Flow cytometry

For the analyses of different intratumoral subpopulations, we used specific multicolor panels for flow cytometry. The following markers were used to stain memory subpopulations of CD4^+^ and CD8^+^ T cells: CD45 APC-Cy7, CD3 Alexa Fluor 700, CD4 PE-CF594, CD8 Pacific Blue, CD45RO APC, CCR7 FITC, CD62L PE-Cy7 and CD95 PE-Cy5. For the phenotype of myeloid dendritic cells (DCs) and plasmacytoid DCs, the following antibodies were used: CD45 APC-Cy7, CD123 PE-Cy5, CD303 PE-Cy7, CD304 PE, CD40 Alexa Fluor 700, CD86 PE Dazzle-594, HLA-DR FITC, CD1c Brilliant Violet 421 and CD141 APC. For the analysis of MDSCs, the following antibodies were combined: CD45 APC-Cy7, CD33 APC, CD66b PE, CD14 PE-Cy7, CD11b Brilliant Violet 421 and HLA-DR FITC. For the Treg population, CD45 APC-Cy7, CD3 Alexa Fluor 700, CD4 PE-CF594, CD25 PE, CD127 Brilliant Violet 421 and FoxP3 Alexa Fluor 647 antibodies were used. Finally, to determine the follicular T cell population, the following antibodies were used: CD45 APC-Cy7, CD200 PE, CD3 Alexa Fluor 700, CD4 PE-CF594, PD-1 Brilliant Violet 421 and CXCL13 APC. Briefly, cells were stained with LIVE/DEAD Fixable Aqua for 20 min in dark conditions at room temperature. After washing with PBS containing 2% FBS, the cells were stained for 30 min at 4 °C in the dark with surface antibodies according to the designed multicolor panels. For intracellular staining, the cells were fixed, permeabilized and stained with anti-FoxP3 and anti-CXCL13 for the Treg and follicular T cell multicolor panels, respectively. Then, the cells were acquired by flow cytometry using a FACSAria IIU flow cytometer (BD Immunocytometry Systems, San José, CA, USA), and the results were subsequently analyzed using FlowJo v10.8.1 software (BD Life Sciences, https://www.flowjo.com/).

### Immunohistochemistry

Formalin-fixed, paraffin-embedded tissue sections from 8 BCP and 6 HD were stained for CD3, CD4, CD8, CD20, CD23, CD45, CD68 and FoxP3 markers by IHC. Tissue sections were deparaffinized in xylene and rehydrated with decreasing concentrations of ethanol. Antigen retrieval was performed at 90 °C for 5 min using pH 9.0 EDTA buffer (CD3, CD4, CD8, CD20, CD23, CD45, CD68, FoxP3). Endogenous peroxidase activity was inhibited using EnVision FLEX Peroxidase-Blocking Reagent (DAKO, Agilent, Santa Clara, USA, SM801). Then, tissue sections were incubated with primary monoclonal antibodies against CD3 (DAKO, IR503), CD4 (DAKO, IR649), CD8 (DAKO, IR623), CD20 (DAKO, IR604), CD23 (DAKO, IR781), CD45 (DAKO, IR751), CD68 (DAKO, IR613), and FoxP3 (Abcam Inc., ab20034). The sections were then rinsed with Envision FLEX Wash Buffer (DAKO, Agilent). Following washing, the sections were overlaid with Envision FLEX/HRP (DAKO, Agilent, SM802) and incubated for 30 min at RT. The immunohistochemical reaction was developed with a 3,3-diaminobenzidine tetrahydrochloride (DAB) solution, EnVision FLEX DAB + Chromogen (DAKO, Agilent, SM803). The sections were counterstained with Harris's hematoxylin, dehydrated, coverslipped, and observed under an optical microscope. Positive and negative controls were performed and validated for each antibody.

### Pathological assessment

For the evaluation of TILs, stromal and intratumoral sections were observed under low magnification (× 4) to determine the type of inflammatory infiltrate and the frequency of stromal TILs (sTILs). For the other markers, two different histological areas of each tissue, including invasive and stromal parts, were examined. Five high-magnification views (40 ×) were randomly selected to determine the frequency of positive cells and determine the mean values of positivity. Microscopic analyses were evaluated independently by two investigators who had no prior knowledge of the clinical data. Discrepancies between the two observers were reviewed jointly with a third trained BC pathologist to reach consensus.

### T-cell receptor (TCR) sequencing and analysis

TCRB sequencing was performed on genomic DNA purified from PB (n = 4), tumor (n = 8) and normal breast tissue (n = 3). TCR beta chain CDR3 regions were sequenced by immunoSEQ (Adaptive Biotechnologies, Seattle, WA) with primers annealing to V and J segments, resulting in amplification of rearranged VDJ segments from each cell. Sequencing was performed on an Illumina HiSeq system (Illumina, San Diego, CA). T cell density, Simpson clonality and diversity values were obtained through the analyzer website.

### T cell stimulation

A total of 5 × 10^6^ PBMCs/mL from BCP pre- and post-NAT were stimulated with anti-CD3/CD28/CD2 microbeads (T Cell Activation/Expansion Kit, human, Miltenyi Biotec). Then, T cell activation was evaluated by flow cytometry through CD3 and TCR internalization, CD69 and CD25 expression, and phosphorylation of ZAP70, mTOR, and AKT, along with markers for memory subpopulations (CCR7 and CD45RO). Cells were acquired by flow cytometry using a FACSAria II flow cytometer (BD Immunocytometry Systems, San José, CA, USA), and the results were subsequently analyzed using FlowJo v10.8.1 software.

A manual analysis was performed followed by an automated analysis in the live lymphocyte gate. Single live CD3^+^ cells for each file were exported and concatenated for analysis by tSNE dimensionality reduction using flowSOM v2.6 in FlowJo software v10.8.1 followed by a comparison of each sample in the concatenated file to identify the proportions of each group and the corresponding phenotype. Finally, the same region of live lymphocytes was exported to a file in FCS to be analyzed with the CITRUS algorithm implemented in R software v3.6.3 (https://www.r-project.org/), allowing the identification of significant differences between groups of patients from pooled populations ex vivo and after in vitro stimulation.

### Statistical analysis

Statistical analysis of the significance between two groups was calculated using the Mann–Whitney U test. Differences among subject groups were evaluated using Kruskal–Wallis and Dunn’s posttest for multiple comparisons. For all cases, the differences were considered statistically significant when *p* < 0.05. GraphPad Prism v9.3.1 for Mac OS X (GraphPad Software, La Jolla California USA, www.graphpad.com) was used for the statistical analyses.

## Results

### Study population

Thirty-three BCP and 10 HD were included in the study. The experimental design used can be seen in Fig. 1. The numbers of patients in stage I were (n = 2), stage II (n = 20) and stage III (n = 11). Estrogen receptor (ER), progesterone receptor (PR), *Her2* expression and Ki-67 percentage were used to classify the samples as follows: luminal A (n = 11), luminal B (n = 15), triple-negative (TN) (n = 5) and *Her2* (n = 2). All BCP received NAT before surgery. Received NAT regimens are shown in Table 1. The patients’ ages ranged from 29 to 79 years; the mean age at diagnosis was 53.9 ± 1.6 years. Among the 33 BCPs analyzed, 9 (27.3%) achieved a pathologic complete response (pCR), and 21 (63.6%) patients had PLNs.

### NAT induces changes in the immune microenvironment in tumor tissue

**Figure 2:**
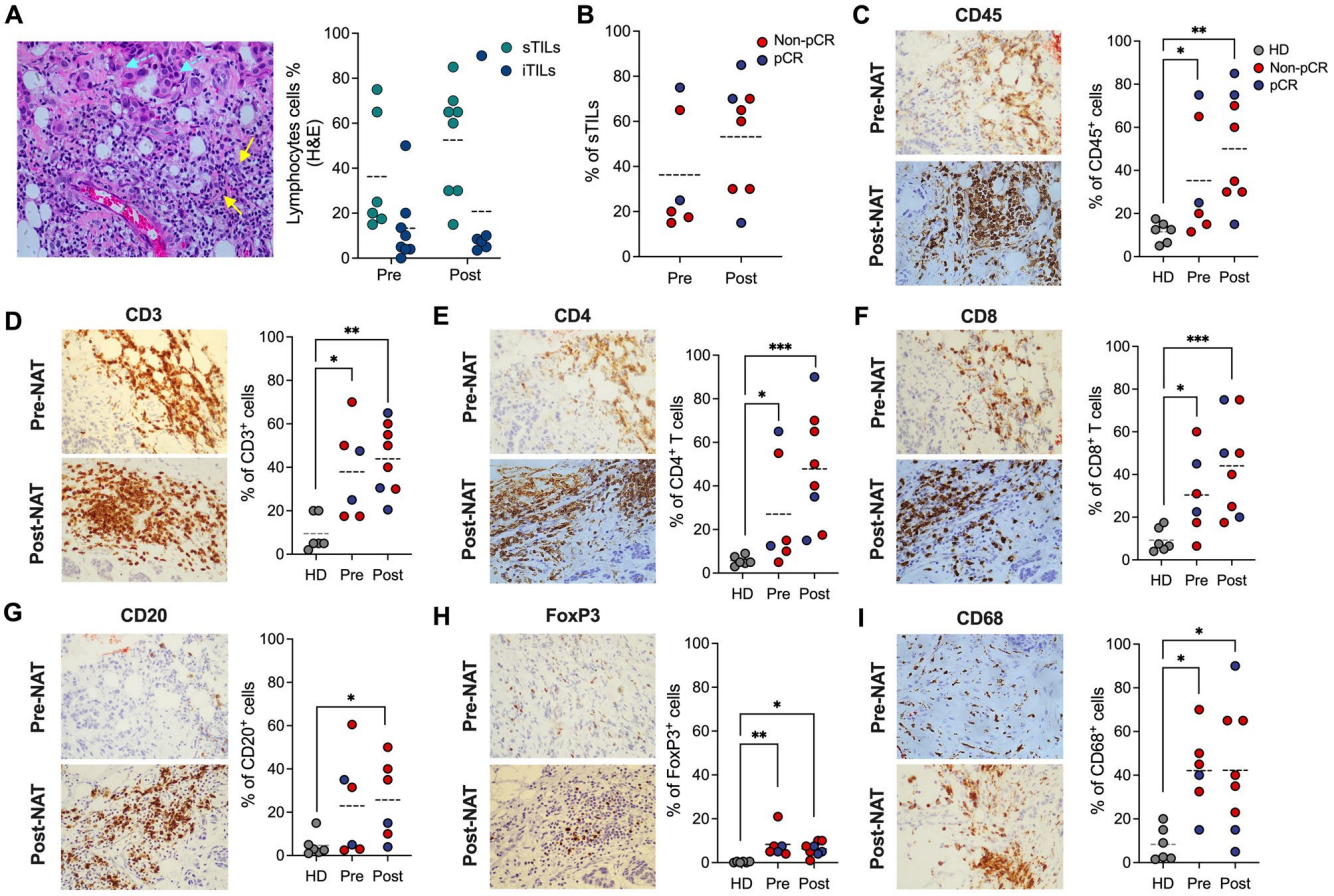
NAT changes the immune microenvironment in breast cancer patients. (**A**) Leukocyte infiltration of tumors from breast cancer patients by hematoxylin and eosin (H&E) staining of tissue sections pre- and post-NAT. Yellow arrows show stromal tumor-infiltrating lymphocytes (sTILs), and celeste arrows show tumor-infiltrating lymphocytes (iTILs). Images are at 40X magnification. (**B**) Frequency of sTILs in BCP with non-pCR or pCR pre- and post-NAT. (**C**) Representative IHC for CD45+  sTIL (left) staining in tumor samples pre- and post-NAT from the same individual and frequency of CD45^+^ cells (right), (**D**) CD3^+^ (**E**) CD4^+^, (**F**) CD8^+^, (**G**) CD20^+^, (**H**) FoxP3^+^, (**I**) CD68^+^ cells. In all cases, each point represents independent samples: gray circles correspond to HD, blue circles to pCR and red circles to non-pCR patients. Data are represented as the mean ± SEM. The *p* values were calculated using a Mann–Whitney test. *p < 0.05, **p < 0.01, ***p < 0.001.

To evaluate the composition of tumor-infiltrating leukocytes in BCP, tumors from 8 BCP (before and after NAT) and 6 HD were evaluated by IHC detection in tissue sections of intratumoral TILs (iTILs) and stromal TILs (sTILs). Figure 2A and supplementary Fig. 1A shows sTIL and iTIL infiltration in H&E-stained sections of tumor tissues. After NAT, an increase in sTIL frequency was observed in BCP with pCR and non-pCR (Fig. 2B). Figure 2C–I shows the results of IHC staining using different markers, such as CD45 (Fig. 2C), CD3 (Fig. 2D), CD4 (Fig. 2E), CD8 (Fig. 2F), CD20 (Fig. 2G), FoxP3 (Fig. 2H) and CD68 (Fig. 2I). BCP increased the frequency of all evaluated markers compared with HD (Fig. 2C–I). Our study found that sTILS, CD45^+^, CD3^+^, CD4^+^, and CD8^+^ cells tended to have a higher frequency in tumor tissue (stromal) and that the frequencies of CD20^+^, FoxP3^+^ and CD68^+^ cells in tumor tissue did not change after-NAT compared with before-NAT.

Furthermore, a positive but not-significant correlation between CD3^+^, CD45^+^, CD4^+^, CD8^+^, CD20^+^ and FOXP3^+^ cells was found in patients before-NAT; however, the same markers showed a non-significant negative correlation with CD68 before-NAT which switched to a positive correlation after-NAT (Supplementary Figure 1B). Likewise, we found a positive but not-significant correlation between the same markers in pCR patients before-NAT but not in non-PCR patients after-NAT (Supplementary Figure 1C).

### The lymphoid immune composition in tumor tissue before-NAT is distinct from normal tissue

**Figure 3:**
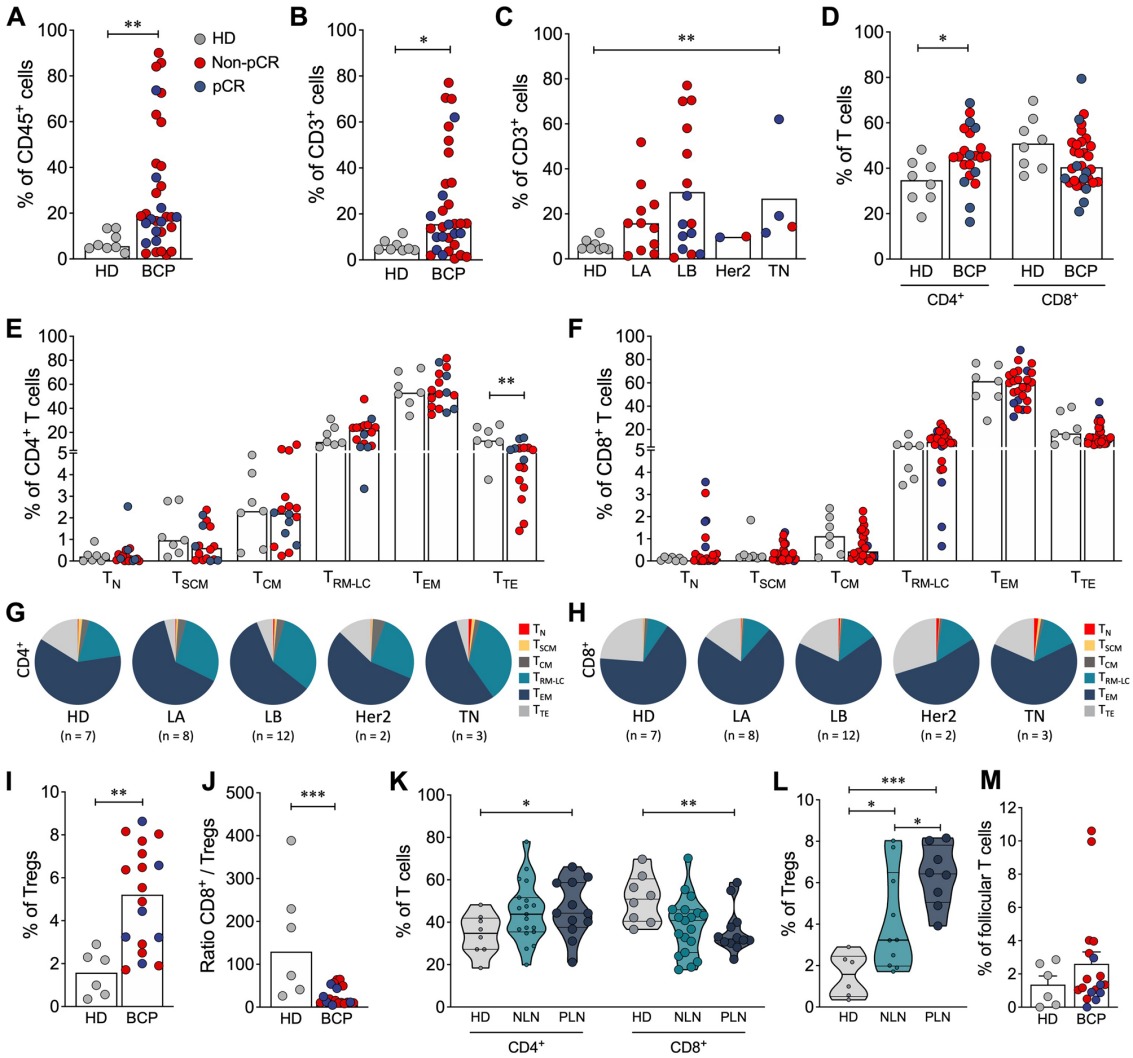
T cell composition in tumor and normal breast tissue. (**A**) Frequency of CD45^+^ cells from healthy donors (HD) and breast cancer patients (BCP). (**B**) Frequency of CD3^+^ cells from HD and BCP. (**C**) Frequency of CD3^+^ cells according to the molecular subtype of breast cancer: luminal A (LA), luminal B (LB), Her2 and triple negative (TN). (**D**) Percentage of CD4^+^ and CD8^+^ T cells. Distribution of memory subpopulations of CD4^+^ (**E**) and CD8^+^ T cells (**F**) evaluated using the CD45RA, CD62L, CCR7 and CD95 markers to define naïve (T_N_), stem cell memory (T_SCM_), central memory (T_CM_), tissue-resident memory-like (T_RM_), effector memory (T_EM_) and terminally differentiated effector (T_TE_) T cells. Pie charts of the distribution of CD4^+^ (**G**) and CD8^+^ T cell (**H**) memory subpopulations according to the molecular subtype of breast cancer. (**I**) Frequency of CD4^+^CD25^+^Foxp3^+^ regulatory T cells (Tregs). (**J**) Ratio of CD8^+^/Treg cells. (**K**) Frequency of CD4^+^ and CD8^+^ T cells from HD and patients with positive lymph nodes (PLNs) or negative lymph nodes (NLNs). (**L**) Treg frequency according to the lymph node state. (**M**) Frequency of follicular T cells from healthy donors (HD) and breast cancer patients (BCP). Data are represented as the mean ± SEM in (**A, B, C, D, E, F, I, and J**). Data are represented as violin plots, and lines indicate quartiles in K and J. The *p* values were calculated using a Mann–Whitney test. *p < 0.05, **p < 0.01, ***p < 0.001.

We evaluated the TME by multicolor flow cytometry in tumor tissues from BCP after-NAT. Thus, BCP patients, including pCR and non-pCR patients, showed higher infiltration of CD45^+^ cells than HD patients (Fig. 3A), as well as a significant accumulation of CD3^+^ cells (Fig. 3B). The frequency of CD3^+^ cells was differential according to the molecular subtype of cancer, finding a significant increase in the group of TN patients compared with HD (Fig. 3C). Within the CD3^+^ population, the frequency of CD4^+^ T cells increased, while CD8^+^ T cells tended to decrease in comparison with normal tissue (Fig. 3D). T cells are described as a highly heterogeneous cell compartment comprising different phenotypes, functional activities, and survival capacities. Accordingly, different markers have been proposed to identify T cell subpopulations^23^. We included CD45RA, CD62L, CCR7 and CD95 to define naïve (T_N_), stem cell memory (T_SCM_), central memory (T_CM_), tissue-resident memory-like (T_RM_), effector memory (T_EM_) and terminally differentiated effector (T_TE_) T cells (Supplementary Figure 2A). The frequencies of CD4^+^ and CD8^+^ T cell memory subpopulations were similar in tumor and normal tissue (Fig. 3 E, F), except for CD4^+^ T_TE_ cells, which were lower in BCP (Fig. 3E). In terms of molecular subtype of cancer, it was found that the distribution of T_N_ cells was higher in TN patients than in the other groups (Fig. 3 G, H). Additionally, according to the pathologic response, the distribution of memory subpopulations of CD4^+^ T and CD8^+^ T cells was significantly different between HD and non-pCR patients, while the distribution of subpopulations was more similar between HD and pCR patients (Supplementary Figure 2B). Analyzing the phenotypical characteristics of CD4^+^ T cells infiltrating tumor and normal tissue, it was found that independent of the pathologic response, there was an increase in the frequency of Tregs (Fig. 3I), characterized by expression of the IL-2 receptor α chain (CD25) and FoxP3 transcription factor (Supplementary Figure 2C). High CD8^+^ T cell infiltration has been correlated with a better prognosis^6^, whereas the accumulation of Tregs in the TME is associated with a worse prognosis^24,25^. We found a markedly decreased ratio of CD8^+^ T cells to Tregs in BCP compared with HD (Fig. 3J and Supplementary Figure 2D), suggesting that BCP had a suppressor TME rather than an effector TME. According to the nodal state of BCP, we found an increase in CD4^+^ T cells and a decrease in CD8^+^ T cells in PLN compared with HD patients (Fig. 3K), an increase in Tregs in negative lymph node (NLN) patients but a more marked increase in PLN patients (Fig. 3L), and a decrease in the ratio of CD8^+^ T cells to Tregs in NLN and PLN patients (Supplementary Figure 2D). These results suggested that the dysregulation of the immune response, with more suppressive than effector activity, favored the migration of tumor cells to the lymph nodes. T follicular helper cells (Supplementary Figure 2E) are a specialized subset of CD4^+^ T cells with prognostic significance in the tumor^26^; however, no differences were found in the frequency of these cells between HD and BCP (Fig. 3M).

### Breast cancer patients after-NAT have an increased frequency of PMN-MDSC-LC compared to HD

**Figure 4:**
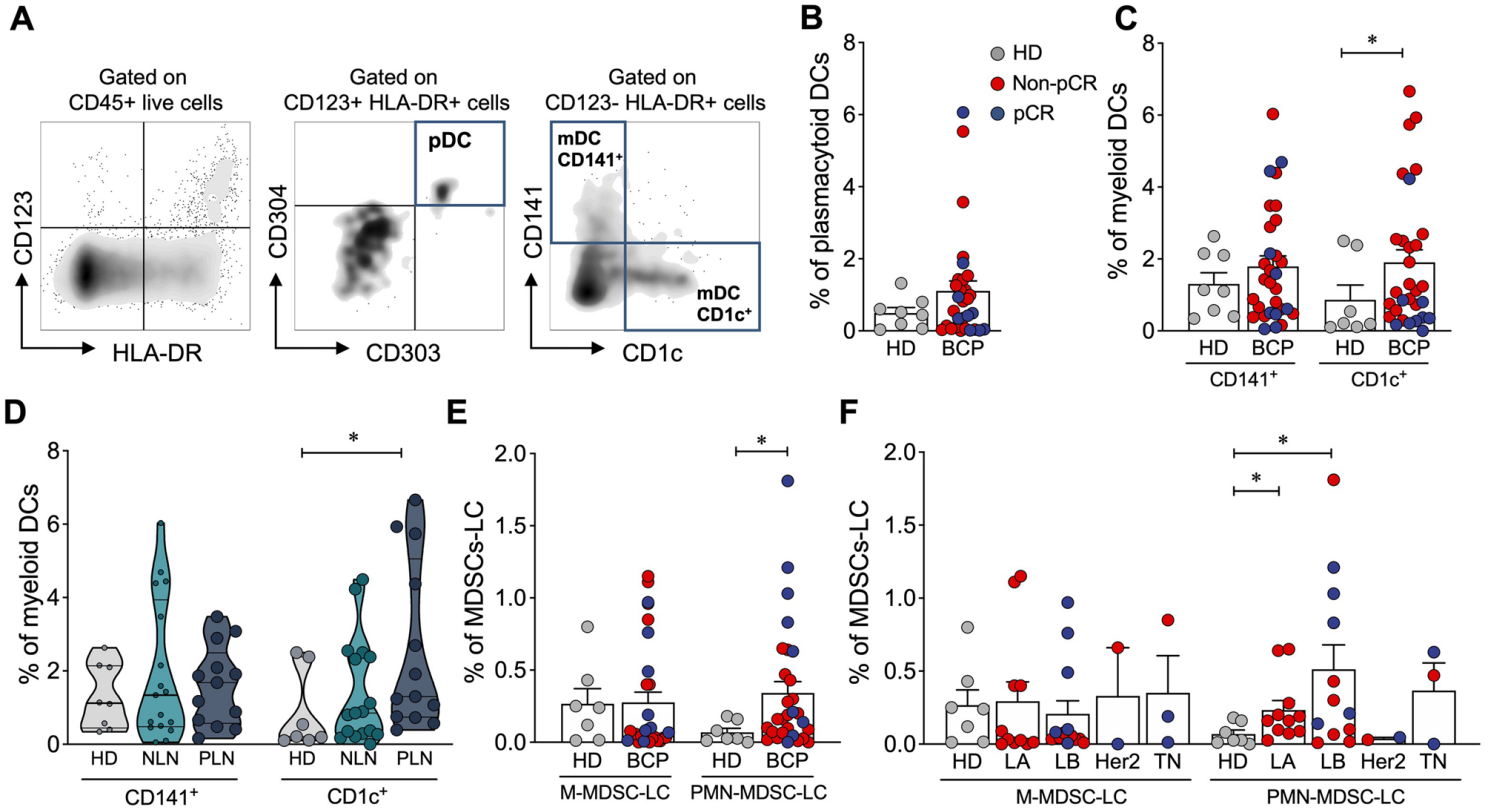
Immune composition in tumor and normal breast tissue. (**A**) Representative FACS analysis of plasmacytoid dendritic cells (pDCs) and CD141^+^ and CD1c^+^ myeloid dendritic cells (mDCs). (**B**) Frequency of pDCs from HD and BCP. (**C**) Percentage of CD141^+^ and CD1c^+^ myeloid DCs. (**D**) Frequency of CD141^+^ and CD1c^+^ myeloid DCs from HD patients and patients with positive lymph nodes (PLNs) or negative lymph nodes (NLNs). (**E**) Frequency of monocytic MDSCs (M-MDSCs) and polymorphonuclear MDSCs (PMN-MDSCs). (**F**) Frequency of M-MDSCs and PMN-MDSCs according to the molecular subtype of breast cancer: luminal A (LA), luminal B (LB), Her2 and triple negative (TN). In all cases, each point represents independent samples: gray circles correspond to HD, blue circles to pathologic complete response (pCR) and red circles to non-pCR patients. Data are represented as the mean ± SEM. The *p* values were calculated using a Mann–Whitney test. *p < 0.05, **p < 0.01, ***p < 0.001.

Dendritic cells (DCs) encompass plasmacytoid DCs (pDCs) and two subsets of myeloid DCs (mDCs): CD1c^+^ mDCs and CD141^+^ mDCs (Fig. 4A). pDCs, cells that contribute to the immunosuppressive tumor microenvironment, and CD141^+^ mDCs, cells that play a significant role in antigen cross-presentation, were found in the same proportion in tumor and normal tissue (Fig. 4 B,C). Conversely, CD1c^+^ mDCs, cells with an inferior capacity to cross-present antigen to CD8^+^ T cells compared with CD141^+^ DCs, were increased in tumor tissue, but the differences were at the expense of non-pCR patients (Fig. 4D) and PLN patients (Fig. 4D). MDSC-like cells (Supplementary Figure 2F) are another population that is involved in an immunosuppressor TME. We found no differences in the frequency of monocytic MDSC-like cells (M-MDSC-LC) between HD and BCP, but we found an increase in the frequency of polymorphonuclear myeloid-derived suppressor like cells (PMN-MDSC-LC) in BCP compared with HD (Fig. 4E), and this difference appeared to be at the expense of LB tumor samples (Fig. 4F).

### T cell activation in peripheral blood after NAT

**Figure 5:**
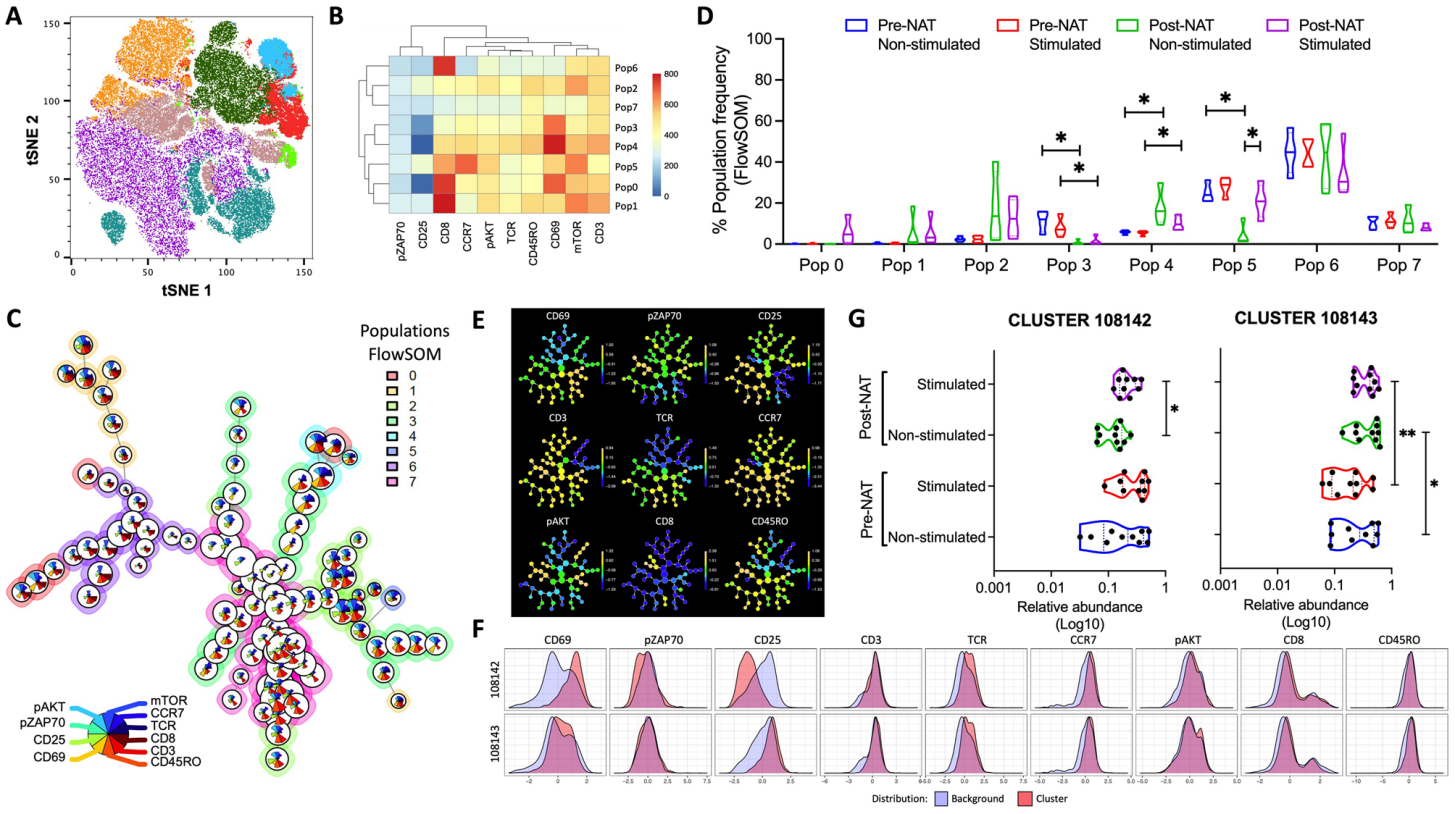
Response of PBMCs to in vitro stimulation of patients before and after NAT. (**A**) t-SNE plot for PBMCs with FlowSOM-based clusters. (**B**) Heat map indicating the expression level of different markers used in flow cytometry for each of the populations determined by FlowSOM grouped by their hierarchical proximity. (**C**) Spanning tree of the subpopulations determined by FlowSOM with the expression level of each marker within the circle (the size of the circle is proportional to the number of events). (**D**) Frequency of each FlowSOM subpopulation in the four groups of patient samples. (**E**) CITRUS-based expression tree for each of the markers in each subpopulation. (**F**) Histograms of expression of the clusters that differed statistically between the groups of samples. (**G**) Relative abundance (Log10) of the clusters that significantly differentiate the groups of patient samples. *p < 0.05, **p < 0.01.

Additionally, we evaluated T cell activation in a cohort of BCP pre- and post-NAC in PB. Initially, the flow cytometry data were analyzed with the FlowSOM algorithm on a tSNE dimension reduction map, where 8 main subpopulations were established (Fig. 5A). Using a heat map, the expression level of each marker and their hierarchical relationship were determined, as well as under the spanning tree distribution (Fig. 5B,C). When comparing the distribution of the populations between the groups of patients (stimulated or not in vitro), significant differences were found in populations 3, 4 and 5 (characterized by a high level of CD69 expression), and a significant increase was found in population 4 post-NAT and after stimulation (Fig. 5D). Additionally, when the data were analyzed by CITTRUS, there were two models capable of differentiating the four population groups analyzed (clusters 108142 and 108143) that had an activation phenotype (Fig. 5E,F), similar to that determined in FlowSOM with high expression of CD69 in a cluster with increased CD25 expression. Finally, when comparing the relative abundance of the clusters in the four groups, it was observed that cluster 108143 was increased in after-NAT samples (both stimulated and unstimulated) compared with the pre-NAT samples (Fig. 5G). The above data showed that in response to in vitro stimulation, T cells from PBMCs from post-NAT patients had an increased proportion of activated cells compared with the same patients prior to treatment.

### More T cell clonality and low diversity after NAT treatment

**Figure 6:**
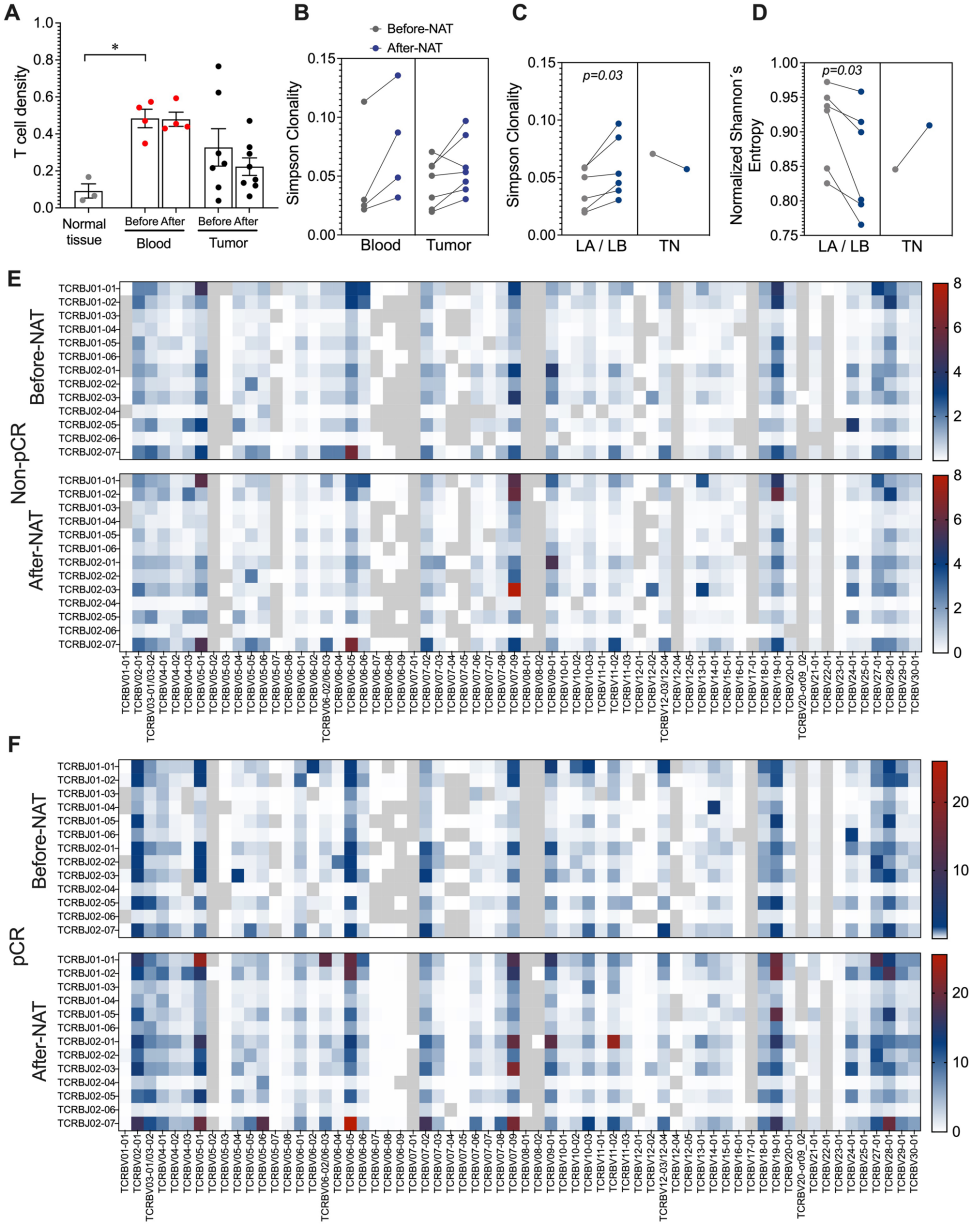
T cell repertoire in blood, tumor, and normal breast tissue before and after NAT. (**A**) The T cell density in each sample calculated by normalizing TCR template counts to the total amount of DNA usable for TCR sequencing; each point represents independent samples. (**B**) Simpson clonality as a measurement of the immune repertoire in blood and tumor samples pre- and post-NAT. (**C**) Simpson clonality in tumor samples pre- and post-NAT according to molecular subtypes of breast cancer. (**D**) Normalized Shannon’s entropy as an index of diversity in each tumor according to molecular subtypes of breast cancer. (**E**) Heat map showing the distribution of clones in nonpathologic complete response (pCR) patients before and after treatment. (**F**) Heat map showing the distribution of clones in pCR patients before and after treatment. Heat map was created using GraphPad Prism v9.3.1 for Mac OS X (GraphPad Software, La Jolla California USA, www.graphpad.com). The *p* values were calculated using a Mann–Whitney U test. *p < 0.05.

In this study, we used TCRB sequencing to determine the T cell repertoire in samples of PB, tumor, and normal breast tissue from 10 BCP and 3 HD patients. The size of the TCRB repertoire was different for each tissue. The number of T cells in each sample, represented by productive templates, was higher in PB, followed by tumor and normal breast tissue, for both pre- and post-NAT samples (Supplementary Figure 3A). Likewise, tumors tended to have a higher density of infiltrating T cells than normal breast tissue, with both containing a lower density of T cells than that observed in PB (Fig. 6A). The clonality in PB and tumors post-NAT was higher than that in pre-NAT samples (Fig. 6B and Supplementary Figure 3B), except for one patient who was classified as TN (Fig. 6C and Supplementary Figure 3C), suggesting a possible expansion of specific clones of T cells, unlike in the TN patient (Supplementary Figure 4A,B). This phenomenon was verified by measurement of the normalized Shannon´s entropy, which showed a lower diversity in LA and LB patients after NAT (Fig. 6D and Supplementary Figure 4C,D). The patients showed a higher frequency of clones than HD patients (Supplementary Fig. 3D). In terms of pathologic response, in non-pCR (Fig. 6E) and pCR (Fig. 6F) patients, the expansion of some specific clones was observed after NAT; however, in pCR patients, expansion was more marked (Fig. 6F), suggesting that these clones might play an important role in the control of the disease.

## Discussion

To date, various NAT regimens have been tested and implemented, providing a favorable outcome in primary tumors and reducing the risk of progression. However, there is a large amount of evidence of the ability of chemotherapy to increase the risk of cancer progression by different mechanisms, involving the induction of stress and the transformation and maintenance of an intratumoral inflammatory microenvironment. These changes favor the emergence of chemotherapy-resistant tumor cells promoting tumor invasiveness^27^.

In the present study, we performed a detailed characterization of the immune response generated after NAT in luminal A, luminal B, basal and HER2+ BCP and evaluated the relationship with the pathological response and clinical parameters related to tumor control. Understanding the type of immune response induced after NAT and its relationship with the progression of the disease can shed light on suitable therapeutic targets not only to treat the primary tumor but also to induce effective control of metastases. The starting hypothesis was that adaptive immune response activation with a preferential expansion of T cells could be related to a better response to NAT and even to pCR. To answer these questions, we evaluated the immune infiltrate distribution by IHC and flow cytometry and the TCR rearrangements present before and after NAT by TCR sequencing. T cell activation in PB was also studied in some patients, and all the parameters related to the clinical and pathological responses were analyzed.

We observed a baseline tumor immune infiltrate in most of the patients, which increased after NAT, both for CD4^+^ and CD8^+^ T cells and for B cells. CD8^+^ stromal localization was associated with pCR as previously reported^28^. Clonal expansion and early activation of peripheral T cells evidenced after NAT and related to pCR responses highlight the role of the adaptive immune response in tumor control.

In fact, the relationship between the adaptive immune response and a better evolution of the tumor was reported^29^, but it was only until recently that the dynamics of the immune response and its relationship with chemotherapy and the molecular subgroups of BC began to be understood. In fact, the grade and type of stromal versus intratumoral infiltrate is a prognostic marker of the response to adjuvant and NAT, as well as control of metastases, mainly in TN and HER2+ breast cancers. However, the lymphoid infiltrate is not always effective due to the occurrence of suppressive TME^30^.

While the role of CD8^+^ cells in the tumor stroma has been related to direct tumor destruction, the role of other immune cells is more ambiguous. We found a negative correlation between intratumoral CD68^+^ macrophages and Tregs, with the lack of pCR; however, a larger number of patients must be studied to confirm this finding. Tumor-associated macrophages (TAMs) are tissue-resident differentiated monocytes with phagocytic activity and are conventionally classified into M1 and M2 subtypes depending on their differentiation status and functional role. M1 macrophages are characterized by their proinflammatory properties, thereby promoting antitumor Th1-type responses; M2 macrophages are instead anti-inflammatory in nature and secrete IL-10, transforming growth factor-β (TGF-β), and other mediators favoring the establishment of a tolerogenic microenvironment, as well as proangiogenic factors. CD68 is used as a prototype macrophage marker, but it does not discriminate between M1 and M2 macrophages; however, it continues to be used in conventional pathology, explaining the ambiguous results observed when relating CD68 to the cancer response and pCR^31^. It is possible that Tregs favor the differentiation of highly plastic CD68 cells in M2 macrophages because of the generation of immunosuppressed microenvironments, as recently suggested^28^.

A significant body of research relates a high infiltration of CD68 TAMs in the BC microenvironment with an unfavorable outcome^32^. High TAMS has been linked to reduced survival, high tumor grade, larger tumor size, and TN phenotype^33^. TAMS has been shown to enhance tumor progression through the promotion of tumor cell proliferation, angiogenesis, motility, and extravasation of tumor cells and to suppress T cell function^34^. However, in contrast with their protumor effects, CD68^+^ TAMs can also exhibit tumoricidal properties. It has been demonstrated that some chemotherapeutic agents exert their anticancer effects through the tumor killing actions of TAMS. TAMS mediate antibody-dependent cellular cytotoxicity, which is the mechanism underlying the anticancer action of monoclonal therapies such as anti HER2/neu therapy^35^. CD68 is part of the 21-gene oncotype profile, where its presence predicts a greater benefit from chemotherapy^36^ and pathologic response^37^. Thus, a significant body of research supports the role of CD68^+^ cells in enhancing the effects of chemotherapy. Conversely, TAMs have also been associated with promoting chemoresistance through a variety of mechanisms, including a misdirected tissue repair response. Further studies, including subsets of TAMs, are needed, for example, a CD163^high^, CD86^low^, IL-10^high^ population for identification of M2 macrophages related to a poor outcome in BC^38^ and reduced survival^39^. In a previous work, it was found that the joint analysis of CD68, with the presence of TILs and the expression of PD-L1, is more appropriate than the determination of TILs alone, particularly when it is analyzed with the expression of ER and HER+, and it is significantly associated with an excellent response to NAT^40^. If we consider our results, the inclusion of Tregs in this multivariable analysis could add more prognostic value.

Otherwise, we observed a significant increase in intratumoral CD45^+^ cells, preferentially CD4^+^ T cells, concomitant with an increase in Tregs and a decrease in the CD8^+^/Tregs ratio. Although no significance was found for BC subtypes, probably due to small number of BCP in Luminal A, HER2 and TN groups, a significant difference was observed between PLN and NLN patients. The protective role of CD8^+^ cytotoxic T cells and CD4^+^ Th1 cells and effective antitumor immunity are unquestionable^6,41^. The deleterious role of the intra-tumoral suppressive response in the control of metastases exerted by Tregs^30^ has been previously shown.

In a recent study, high infiltration of FOXP3 and PD-L1 was associated with HER2 positivity and p53 overexpression and related to invasive carcinoma compared with pure ductal carcinoma in situ^42^. The FOXP3^+^/CD8^+^ T cell ratio was found to be an independent adverse prognostic factor in the hormone receptor-positive subgroup, especially in the luminal A subtype^43,44^. Altogether, these data associate the role of FOXP3 in promoting tumor migration. Furthermore, its presence before chemotherapy is associated with a lower response to NAT, and its increased detection in residual tumors corroborates its role in the generation of metastases in PLN patients.

We also found a significant infiltration of PMN-MDSC-LC in BCP and an increase, although not significant, in M-MDSC-LC. MDSCs are a heterogeneous population of immunosuppressive pro-tumoral leukocytes that result from abnormal myelopoiesis as a consequence, for example, of a pathological condition such as cancer that is accompanied by an increase in ROS, as well as IL-6, among other cytokines. They inhibit antitumor immunity by producing immunosuppressive factors such as arginase, reactive nitrogen and oxygen species and inducing the activation of Tregs^45,46^. There are two major subpopulations of MDSCs, with PMN-MDSCs being the most abundant. The suppressive activity of this population is critically dependent on lipid accumulation^47^. They themselves produce large amounts of ROS that favor the oxidation of the lipids they accumulate, which are involved in reducing the ability of DCs to perform cross priming^48^.

Chemotherapy induces multiple changes in the tumor microenvironment. One of them is the induction of MDSC recruitment, as observed before systemic treatment of BCP with doxorubicin-cyclophosphamide every 14 days. There was also a significant correlation between circulating MDSCs and the clinical cancer stage. In addition, patients with extensive metastatic tumors had the highest percentage and absolute number of MDSCs^49^. In head and neck cancer, the outcome of preoperative cetuximab treatment can be predicted by PMN-MDSC numbers, which decreased in the responder group and remained unchanged in nonresponders^50^. PMN-MDSC frequencies have been related to the presence of tumor metastasis, possibly by priming an organ-specific premetastatic niche^51^.

The presence of PMN-MDSCs could be related to the increase in CD1c mDCs that we observed in PLN patients in our study. In fact, the CD1c (DC-2) population characterized as previously described^52^ is effective in inducing Th2 responses; it does not induce proliferation of allogeneic T cells and is directly related to the suppression of the immune response in cancer^53^. The interesting and worrisome finding is that this population is detected in the tumors of patients undergoing NAT, which suggests that oxidative stress generated by chemotherapy may be a triggering event for increased PMN-MDSC migration. These results may be related to what was found when analyzing the T cell response. In fact, we observed that NAT increases the level of intracellular activation of T cells, which suggests an increase in antigenic presentation. However, when performing the clonality analysis, we found that this activation seemed to generate a preferential clonal expansion in pCR patients. Clonal expansion was observed for some of the clones evidenced before NAT. However, the emergence of new clones in the top 10 frequencies was observed (data not shown), suggesting the presentation of new tumor antigens promoted by NAT, possibly associated with the appearance of intratumoral tertiary lymphoid nodes. This dynamic is evident in all molecular subgroups. It would be very interesting to determine whether these new clones are part of the naïve T cell population and if both CD4^+^ and CD8^+^ cells are observed in patients. We are now evaluating in detail the interindividual differences of these expansions in a larger group of patients undergoing NAT to determine if specific patterns are related to the control of metastases.

In overall, these results suggest that current treatment schemes could be complemented to achieve a balance in immune subsets improving the activation of the antitumor immune response and, therefore, a better response to treatment.

## Supplementary Information

Supplementary Information.
