# Supplementary material for: The breast cancer immune microenvironment is modified by neoadjuvant chemotherapy
Source: Sci Rep. 2022 May 13;12:7981. doi: 10.1038/s41598-022-12108-5 (PMC9106657; doi:10.1038/s41598-022-12108-5)
Supplement: Supplementary file 1 — Supplementary Information. [file 41598_2022_12108_MOESM1_ESM.pdf]

Supplementary Figure Legends

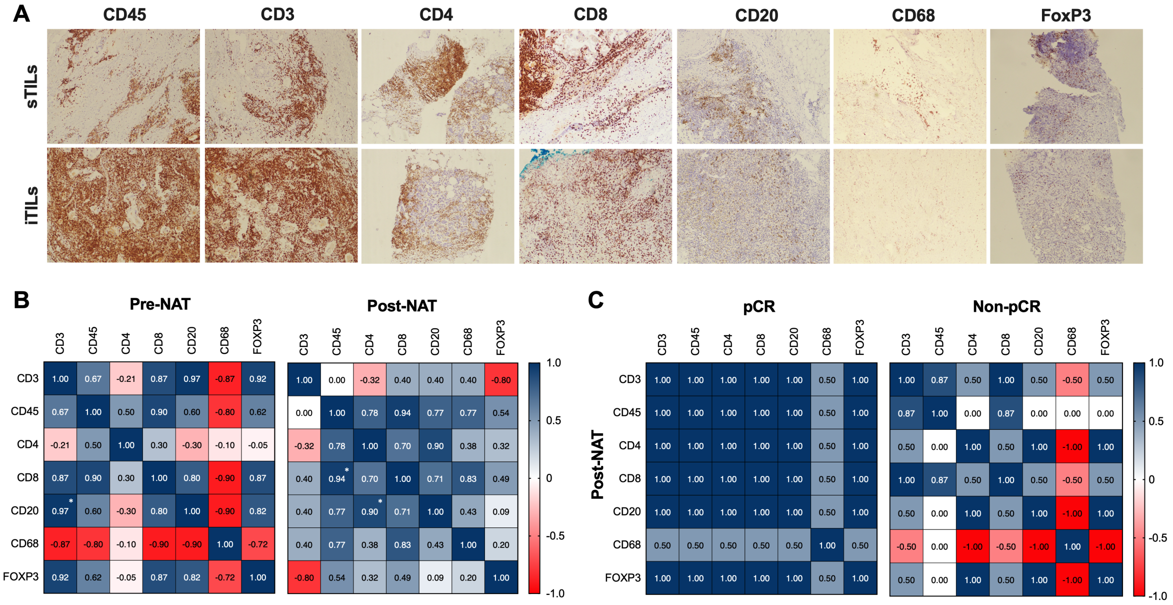

Supplementary Figure 1. Representative images of IHC from tumor tissue and correlations between immune cells and NAT. A. Leukocyte infiltration of tumors from breast cancer patients by hematoxylin and eosin (H&E) staining of tissue sections in stromal tumor-infiltrating lymphocytes (sTILs) and intratumoral tumor-infiltrating lymphocytes (iTILs). Images are at 10X magnification. **B.** Heatmap representation of immune population correlations evaluated in pre- (n=6) and post-NAT (n=8) tumor samples. **C.** Heatmap representation of immune population correlations evaluated in tumor samples post-NAT from pathologic complete response (pCR) (n=5) and non-pCR patients (n=3). *$p < 0.05$.

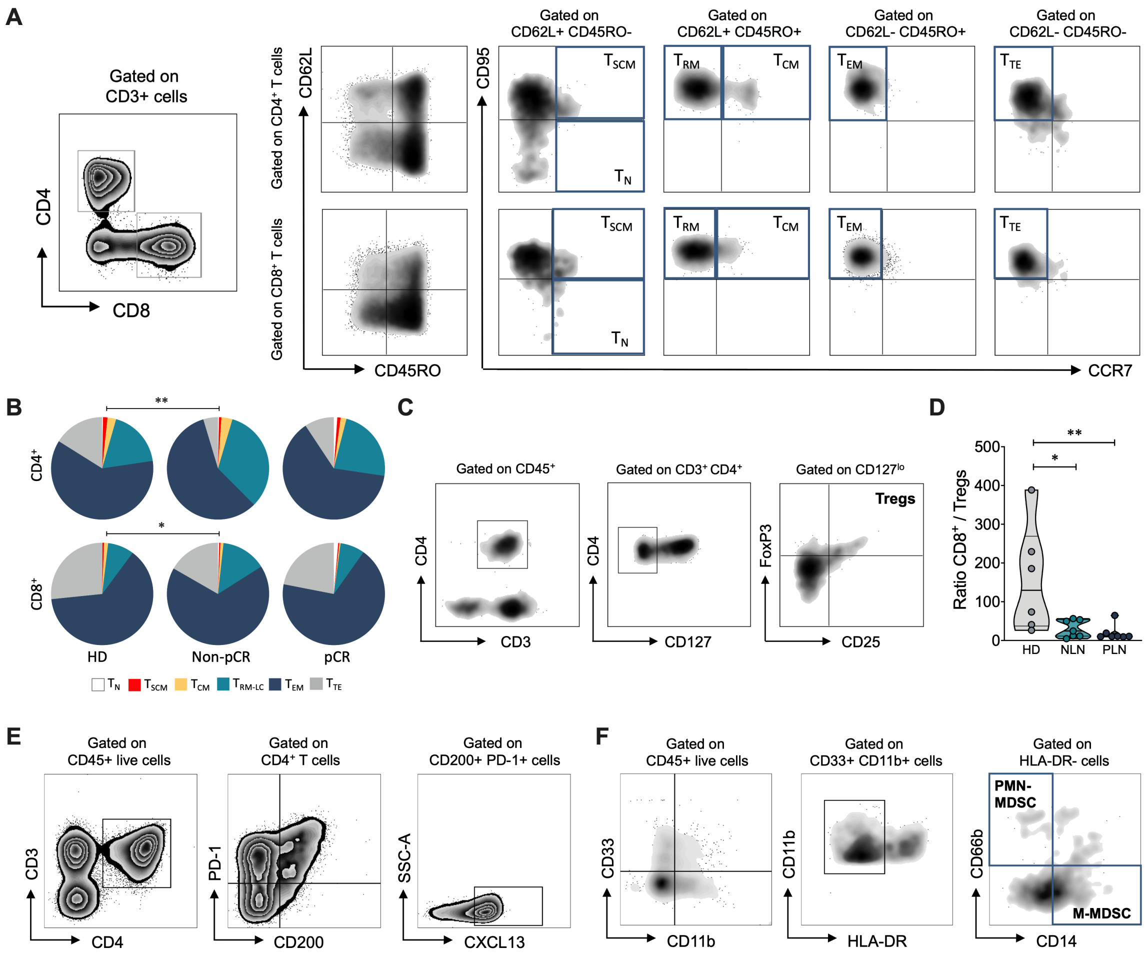

Supplementary Figure 2. Analysis of immune composition in tumor and normal breast tissue. A. Representative FACS analysis of CD4$^+$ and CD8$^+$ memory subpopulations using the CD45RA, CD62L, CCR7 and CD95 markers to define naïve (T_N), stem cell memory (T_{SCM}), central memory (T_{CM}), tissue-resident memory-like (T_{RM}), effector memory (T_{EM}) and terminally differentiated effector (T_{TE}) T cells. **B.** Pie charts of the distribution of CD4$^+$ and CD8$^+$ T cell memory subpopulations from healthy donors (HD), pathologic complete response (pCR) and non-pCR patients. **C.** Representative FACS analysis of regulatory T cells (Tregs). **D.** Ratio of CD8$^+$/Treg cells from HD and patients with positive lymph nodes (PLNs) or negative lymph nodes (NLNs). Representative FACS analysis of follicular T cells **(E),** monocytic MDSCs (M-MDSCs) and polymorphonuclear MDSCs (PMN-MDSCs) **(F)**. The

p values of the permutation test **(B)** are shown in the pie charts. The *p* values in **D** were calculated using a Mann-Whitney U test. *$p < 0.05$, **$p < 0.01$.

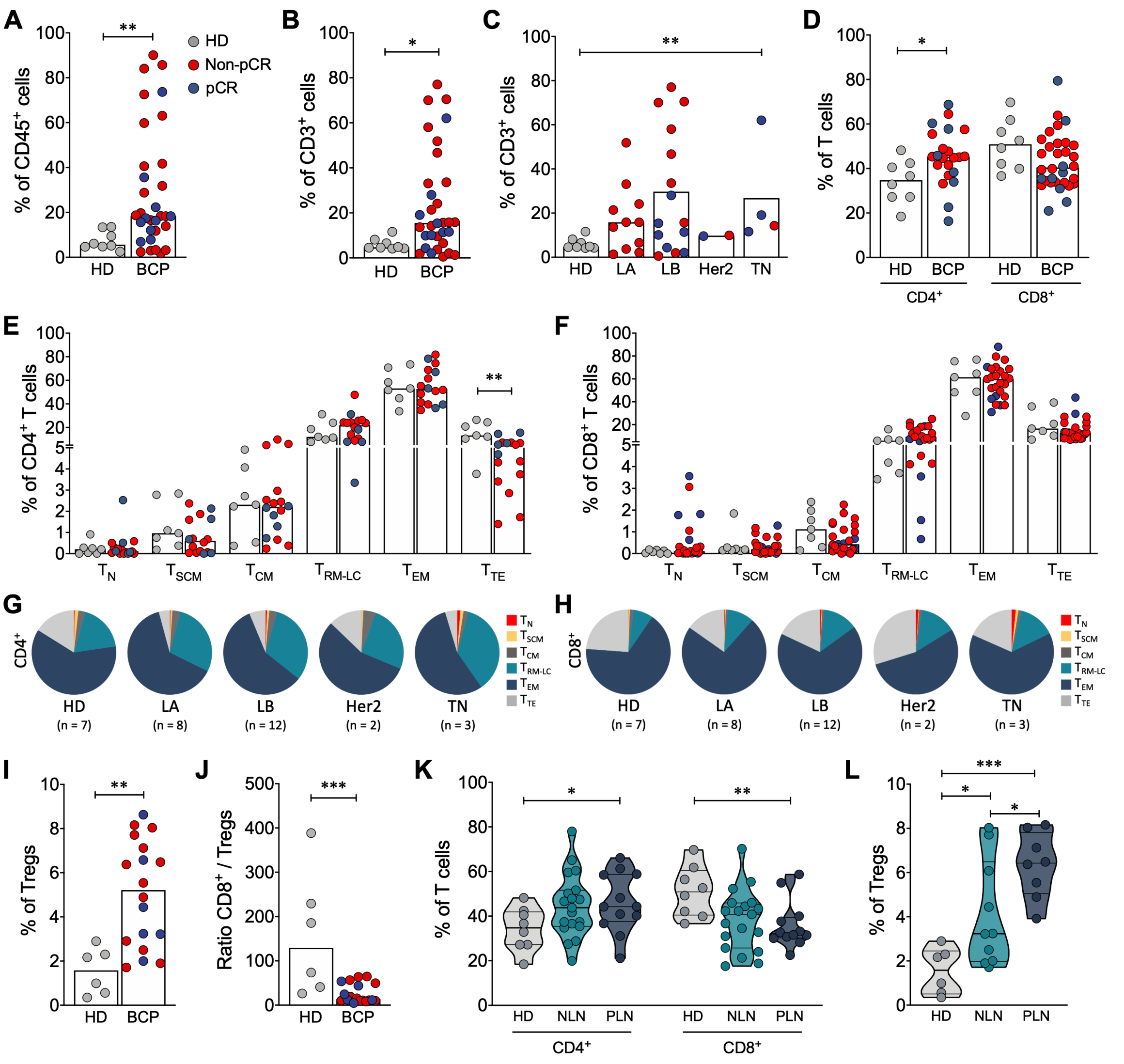

Supplementary Figure 3. Clonality of T cells. A. Productive templates or number of T cells in each sample; each point represents independent samples. **B.** Clonality in blood and tumor samples pre- and post-NAT. **C.** Clonality in tumor samples pre- and post-NAT according to molecular subtypes of breast cancer: luminal A (LA), luminal B (LB), and triple negative (TN). **D.** Heatmap showing the distribution of clones in healthy donors and pre- and post-NAT patients. The *p* values were calculated using a Mann-Whitney U test. $*p < 0.05$.

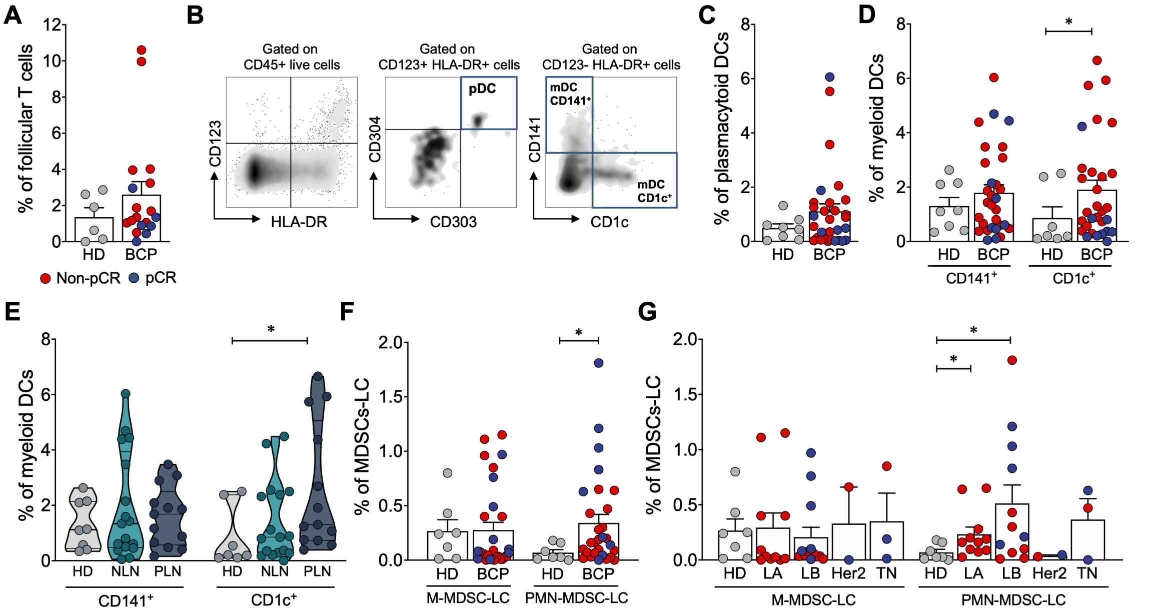

Supplementary Figure 4. Representative analysis of the clonality and diversity of T cells. A. Normalized Shannon's entropy as an index of diversity in a triple-negative patient pre- and post-NAT. **B.** Heatmap of the frequency of T cell clones from triple-negative patients pre- and post-NAT. **C.** Normalized Shannon's entropy as an index of diversity in a luminal B patient pre- and post-NAT. **D.** Heatmap of the frequency of T cell clones from a luminal B patient pre- and post-NAT.
